# Gibbs Sampling Detection for Large MIMO and MTC Uplinks with Adaptive Modulation

**DOI:** 10.3390/s22041309

**Published:** 2022-02-09

**Authors:** Francisco Rosário, Francisco A. Monteiro

**Affiliations:** 1 Instituto de Telecomunicações, 1049-001 Lisbon, Portugal; francisco.rosario@tecnico.ulisboa.pt; 2 Department of Information Science and Technology, ISCTE - Instituto Universitário de Lisboa, 1649-026 Lisbon, Portugal

**Keywords:** Gibbs sampling, large MIMO detection, adaptively modulated MIMO, MTC

## Abstract

Wireless networks beyond 5G will mostly be serving myriads of sensors and other machine-type communications (MTC), with each device having different requirements in respect to latency, error rate, energy consumption, spectral efficiency or other specifications. Multiple-input multiple-output (MIMO) systems remain a central technology towards 6G, and in cases where massive antenna arrays or cell-free networks are not possible to deploy and only moderately large antenna arrays are allowed, the detection problem at the base-station cannot rely on zero-forcing or matched filters and more complex detection schemes have to be used. The main challenge is to find low complexity, hardware feasible methods that are able to attain near optimal performance. Randomized algorithms based on Gibbs sampling (GS) were proven to perform very close to the optimal detection, even for moderately large antenna arrays, while yielding an acceptable number of operations. However, their performance is highly dependent on the chosen “temperature” parameter (TP). In this paper, we propose and study an optimized variant of the GS method, denoted by triple mixed GS, and where three distinct values for the TP are considered. The method exhibits faster convergence rates than the existing ones in the literature, hence requiring fewer iterations to achieve a target bit error rate. The proposed detector is suitable for symmetric large MIMO systems, however the proposed fixed complexity detector is highly suitable to spectrally efficient adaptively modulated MIMO (AM-MIMO) systems where different types of devices upload information at different bit rates or have different requirements regarding spectral efficiency. The proposed receiver is shown to attain quasi-optimal performance in both scenarios.

## 1. Introduction

Wireless networks are heading towards their 6th generation (6G), shifting from the scenario where most users were mobile phones, to one where a paraphernalia of sensors and other devices will lead to pervasive machine-type communications (MTC), which will constitute the dominant type of wireless links [1,2,3]. Many of these devices will be extremely power constrained, with some of them relying on wireless energy transfer to operate [4], and therefore they can only transmit up to a certain power, or during some maximum time during which they have to transmit a short block of data in the shortest time possible to signal an event. These situations imply that at any given time, different devices will be using different modulation orders, which will have to be taken into consideration by the central receiver. On top of that physical-layer, modern coding schemes based on fountain codes or different flavors of network coding can be used to facilitate the data collection [5]. Most proposals for the next generation rely on the existence of a massive MIMO base-station (BS) [6] (today widely considered to be systems with larger than 64 antenna elements [7], (p. 155)) or cell-free networks [8], and they rely on asymmetric MIMO cases, where the number of terminals is much lower than the number of antenna elements at the BS (or the number of receiving antenna elements in a cell-free network). The conditions for such types of operation may not always exist, for example in heavily loaded cells (with a number of terminals approaching the number of antennas at the BS), or when the dimensions of the antenna arrays are physically constrained, particularly if using low-frequency carriers.

In the case of an uplink MIMO channel, when the number of (single-antenna) users *M* is far less than the number of BS antennas *N*, linear processing techniques, such as zero-forcing (ZF) or minimum mean-square error (MMSE), attain near-optimal performance [9], and detectors based on Neumann series expansions can further reduce that complexity [10]. However, when M→N, i.e., as the ratio of the number of antenna elements on both sides of a MIMO system gets closer to 1, linear detection is well-known to severely degrade [11,12]. It is therefore important to use detection methods that perform close to maximum likelihood (ML), i.e., exhaustive search, while yielding low complexity. Despite being optimal, ML and sphere decoding detection (SD) methods are prohibitive in large dimensions due to their exponential complexity [13]. Other possible alternatives are lattice-reduction-aided (LRA) methods [14], punctured SD-based techniques [15], or trellis-based detection approximating the lattice problem by one with a trellis representation [16], all of which hold performance-complexity trade-offs that have been proven to be acceptable for symmetric large MIMO systems.

To address the need to serving devises with widely different requirements (in terms of bit rate, error probability, and spectral efficiency), adaptively modulated MIMO (AM-MIMO) systems, where each user has the possibility of adjusting the utilized modulation scheme according to its channel conditions and rate requirements, are of practical interest [17,18]. The great advantage of these schemes over conventional fixed-MIMO are their high spectral efficiencies, whilst keeping bit error rates (BER) close to a given target [19]. However, finding efficient detection algorithms for such systems has been the focus of some literature; for example, in [20], the authors proposed SD-based methods for optimal decoding of AM-MIMO, even though it is not a scalable solution for large MIMO due to the rising complexity that SD entails.

Advanced statistical methods, such as Markov chain Monte Carlo (MCMC), have already shown significant gains in signal processing for wireless communications. In particular, Gibbs sampling (GS) [21] (section 11-3), which is the particular MCMC technique considered in this paper, can attain ML-like performance, provided the algorithm is run for a sufficient long time [22]. Even so, the conventional GS technique suffers from the so-called “stalling problem”, meaning that for high values of signal to noise ratio (SNR), the BER performance does not improve any further [23]. Gibbs sampling-based techniques have been considered for large symmetric MIMO in recent years [24,25,26,27,28,29]. In this paper, capitalizing on these previous works that use multiple restarts [23,27], we propose a variant of the GS method to perform detection in both large MIMO (with $\frac{N}{M}>0.75$, as in [27]) and AM-MIMO systems in particular, making use of the findings in [22] regarding the adaptation of the so-called “temperature” parameter (TP).

The core idea of the GS technique proposed, and corresponding to the main contribution of this paper, is that at each iteration of the Gibbs sampler, the alternative approach picks one of three possible TP according to a given probability distribution. The TP values and corresponding probability distribution are chosen such that the stalling problem is circumvented, whilst keeping convergence rates high. Numerical results confirm that the proposed variant outperforms both aforementioned works, and that by using a multiple restarts (MR) approach, quasi-optimal performance can be obtained without further increasing processing time.

Following this introductory section, the paper proceeds with the definition of the MIMO channel model in Section 2, which makes use of the mapping of the MIMO communication problem in the complex-domain to one in the real-domain, which can be tackled by the GS method. Section 3 starts by explaining how the GS technique can be applied in solving the MIMO detection problem and shows its pitfalls when a plain GS is used without additional measures; the section then shows how multiple-restarts can be one important additional measure to alleviate those problems. Because the performance obtained via GS detection is highly dependent on the choice of the TP, Section 4 presents some ways of choosing the TP. In Section 5, the central proposal of the paper emerges by probabilistically combining different TP along the iterations of the GS detection process, taking into consideration the observations made in the previous sections. Besides symmetric large-MIMO systems with homogeneous constellations in the antennas, the proposed GS detection is directly extendable to the AM-MIMO systems that naturally arise in MTC scenarios, and that extension is presented in Section 6. In Section 7, we present numerical results that assess the performance of the proposal, and the paper ends by presenting the conclusions in the Section 8.

## 2. System Model

Consider a N×M uplink large MIMO model, where each one of the *M* single antenna devices transmit a symbol from a complex $m_{k}$-QAM alphabet, denoted by $A^{\left(c\right)}$, and where $m_{k}$ corresponds to the constellation size of the *k*th user. The possible transmit symbols by the *k*th user are taken from an $M_{k}$-QAM alphabet $A^{\left(c\right)}$, such that $A^{\left(c\right)}=A+jA$, where $A=\{-\log_{2}\sqrt{M_{k}}+1,\ldots ,-1,1,\log_{2}\sqrt{M_{k}}-1\}$. Let $x_{m}\in A^{\left(c\right)}$ denote the transmitted symbol from user *k* and $x={\left[x_{1},\ldots ,x_{M}\right]}^{T}$ the vector containing the *M* complex symbols. The transmitted symbols from each user (or antenna) are assumed to have normalized energy equal to one, that is $E\{x^{H}x\}=M$, where $x^{H}$ is the Hermitian transpose of the virtual vector transmitted by the terminals. The uncoded transmitted signal x passes through a channel (known at the receiving BS), characterized by the matrix $H\in C^{N\times M}$ and is received by a BS with *N* antenna elements. Thus, the received signal at the base station y can be expressed as:

(1) $$y=gH^{\left(c\right)}x+n,$$

where $g=\sqrt{\frac{\mathrm{SNR}}{M}}$ corresponds to the average gain of the channel and n represents the additive noise at the receive BS. Both entries of H and n are i.i.d. random variables taken from a complex normal distribution CN (0, 1). For implementation purposes, the complex-valued system model is converted into a real-valued one (as in [11] or [23])

(2) $$\left[\begin{array}{c} R\{y\}\\ I\{y\} \end{array}\right]=\left[\begin{array}{cc} R\{H^{\left(c\right)}\} & -I\{H^{\left(c\right)}\}\\ I\{H^{\left(c\right)}\} & R\{H^{\left(c\right)}\} \end{array}\right]\left[\begin{array}{c} R\{x\}\\ I\{x\} \end{array}\right]+\left[\begin{array}{c} R\{n\}\\ I\{n\} \end{array}\right],$$

therefore, designating n=2M=2N, the *real* channel matrix $H\in R^{n\times n}$, while both vectors x, $n\in R^{n\times 1}$ and the complex constellation $A^{\left(c\right)}$ is now a real-valued set of symbols denoted by A.

## 3. Gibbs Sampling for MIMO Detection

### 3.1. Conventional Gibbs Sampling

When applying MIMO detection via GS, a reversible Markov chain is assumed, so that asymptotic convergence to the optimal solution is guaranteed. In other words, if the detector is run for a sufficiently long time, there is a certain positive probability that the optimal solution is visited.

Upon receiving y, the BS starts the detection procedure. Under the assumption that the various transmitted symbols are uncorrelated, retrieving x from (1) can be regarded as a closest vector problem (CVP) in a lattice where the received symbol is one of the lattice points perturbed by a Gaussian distribution [11]. At the initial time instant t=0, an *n*-dimensional starting candidate $x^{\left(0\right)}$ is given to the MCMC detector, which then performs an “ingenious” walk over the real alphabet $A^{n}$ in search of the optimal solution to the following problem:

(3) $$\hat{x}=\arg\min_{x\in A^{n}}||y-gHx||.$$

In particular, the *n*-dimensional lattice formed by $\bar{H}=gH$ (known as the lattice basis) is $\Lambda =\{\bar{H}x:x\in Z^{n}\}$. Then, one can define the Gaussian function centered at $y\in R^{n}$ for standard deviation $\sigma_{n}^{2}=1>0$ as

(4) $$\rho \left(\bar{H}x\right)=\exp\left(-\frac{||y-\bar{H}x{\left|\right|}^{2}}{2}\right),$$

for all $\bar{H}x\in R^{n}$. Hence, the previous expression suggests that the joint probability of interest satisfies

(5) $$p\left(x_{1},x_{2},\ldots ,x_{n}|\bar{H},y\right)\;\propto \;\exp\left(-||y-\bar{H}x{\left|\right|}^{2}\right).$$

From the above expression, the transition rule between consecutive states can be computed. Particularly, it can be regarded as a discrete Gaussian distribution over Λ; hence, sampling techniques such as Gibbs algorithm are applicable. Assume that at time index *t*, the current state is given by $x^{\left(t\right)}\in A^{n}$. Then, at the next time index, the method uniformly picks one random position *j* out of {1,…,n} and computes the conditional probability of transitioning to each of the possible constellation points. With the remaining (n−1) positions fixed, the *j*th index is updated according to a stationary distribution to be detailed in the next subsection.

### 3.2. Gibbs Sampling Implementation

In the GS method herein used, each iteration *t* consists of transitioning each coordinate j=1,…,n to a possible element ω∈A sequentially, based on a sampling rule defined by the following probability mass function (pmf) [23]:

(6) $$p(x_{j}^{\left(t+1\right)}|x^{\left(t\right)},j,y,gH)=\frac{\exp\left(-\frac{1}{2\alpha^{2}}||y-gHx_{j|\omega }{\left|\right|}^{2}\right)}{\sum_{x_{j|\tilde{\omega }}\in A}^{}\exp\left(-\frac{1}{2\alpha^{2}}||y-gHx_{j|\tilde{\omega }}{\left|\right|}^{2}\right)},$$

where $x_{j|\omega }^{T}={\left[x_{1:j-1}^{\left(t+1\right)},\omega ,x_{j+1:n}^{\left(t\right)}\right]}^{T}$ and α is a positive TP. Equation (6) hints that the closer lattice point gHx is to y, the higher the probability that it is is sampled. At the end of each iteration, the ML cost

(7) $$f\left(x^{\left(t+1\right)}\right)≜||y-gHx^{\left(t+1\right)}\left|\right|$$

is computed and compared with the one of the best candidate thus far; then the vector $x^{\left(t+1\right)}$ is fed into the next iteration. After $t_{\max}$ iterations, the algorithm is stopped and the vector yielding the lowest ML cost is the output solution. For further implementation details and complexity reduction techniques of GS see, e.g., [22,27]. The aforementioned process is denoted in the literature by Gibbs sampling, where one iteration involves sampling one entry at a time in the following manner [30,31]:

(8) $$\begin{array}{cc} x_{1}^{\left(t+1\right)} & ∼p(x_{1}|x_{2}^{\left(t\right)},\ldots ,x_{n}^{\left(t\right)},y,\bar{H});\\ x_{2}^{\left(t+1\right)} & ∼p(x_{2}|x_{1}^{\left(t+1\right)},x_{3}^{\left(t\right)},\ldots ,x_{n}^{\left(t\right)},y,\bar{H});\\ \vdots & \\ x_{n}^{\left(t+1\right)} & ∼p(x_{n}|x_{1}^{\left(t+1\right)},x_{2}^{\left(t+1\right)},\ldots ,x_{n-1}^{\left(t+1\right)},y,\bar{H}). \end{array}$$

At the end of each iteration, the ML cost in (7) is computed, and the resulting vector is fed into the next iteration. The algorithm stops after a certain number of iterations and the algorithms’ output is the vector with the best ML cost. The pseudocode for this type of MCMC detector is presented in Algorithm 1.
**Algorithm 1.** Reversible MCMC detector based on Gibbs sampling.**Input:** y,H, initial vector $x^{\left(0\right)}$, number of iterations $t_{\max}$Denote the decision vector by zDenote the ML cost function as f(·)**for**t=1 to $t_{max}$ **do**    Pick a position index j∈{1,2,…,n} from a uniform distribution    Fix the (n−1) symbols of $x^{\left(t\right)}$, and transition the *j*th symbol of $x^{\left(t\right)}$ to ω according to (6)    Denote the new vector by $x^{\left(t+1\right)}$    **if** $f\left(x^{\left(t+1\right)}\right)<f\left(z\right)$ **then**        Update $z=x^{\left(t+1\right)}$    **end if****end for****Output:** z

### 3.3. The Complexity of Gibbs Sampling

For the sake of implementation (to limit complexity), a sequential rather than a reversible MCMC detector will be considered. The only difference resides in the way the updated position index *j* is chosen. In sequential detection, each iteration consists of a block iteration, where the *n* indices are updated sequentially, hence the name.

Denoting the difference at iteration *t* as $d^{\left(t\right)}=y-gHx^{\left(t\right)}$ and in order to further reduce complexity, one may notice that only the *j*th symbol is changed when (6) is computed [22]. Consequently, $d^{\left(t\right)}$ can be expressed as:

(9) $$d^{\left(t\right)}=d^{\left(t-1\right)}-gh_{j}\Delta x_{j|\omega },$$

where $\Delta x_{j|\omega }=x_{j|\omega }^{\left(t\right)}-x_{j|\omega }^{\left(t-1\right)}$ and $h_{j}$ is the *j*th column of H. Therefore, the complexity of computing the norms in (6), when the *j*th symbol is changed, is 2n complex operations (*n* multiplications for each of the products $\left(d^{\left(t\right)}\right){)}^{T}d^{\left(t\right)}$ and $h_{j}\Delta x_{j|\omega }$). Further, and assuming that the number of required iterations will be large (processing for massive MIMO systems), the complexity of an MCMC detector can be reduced even further. This can be achieved by means of a QL factorization of the channel matrix, H=QL and by rewriting (3) as [32]

(10) $$\hat{x}=\arg\min_{x\in A^{n}}\left|\right|\tilde{y}-gLx{\left|\right|}^{2},$$

where $\tilde{y}=Q^{T}y$. Exploiting the fact that L is a lower triangular matrix, the first difference $d^{\left(0\right)}=\tilde{y}-Lx$ costs $n+2\sum_{i=1}^{n}i=n\left(n+2\right)$ multiplication and addition operations (which is lower than the conventional model $2n^{2}$). Moreover, at each iteration, the difference $d^{\left(t-1\right)}-gh_{j}\Delta x_{j|\omega }$ will require $2\left(\frac{n+1}{2}\right)\left(|A|-1\right)$ arithmetic operations. If the conventional model was used, the corresponding complexity would be 2n(|A|−1). Then, and taking into account that computing $\tilde{y}$ yields $O(\frac{2}{3}n^{3}+2n^{2})$, there is a gain in complexity if

(11) $$n\left(n+2\right)+t_{max}\left(n+1\right)\left(|A|-1\right)+\frac{2}{3}n^{3}+2n^{2}<2nt_{max}\left(|A|-1\right)+2n^{2},$$

which holds true when

(12) $$t_{max}>\frac{\frac{2}{3}n^{2}+n+2}{\left(|A|-1)(n-1\right)}n\approx \frac{\frac{2}{3}n^{2}+n}{|A|-1}.$$

Another possibility to keep complexity low, especially in higher constellations (64-QAM and higher), is to only sample one-symbol away neighbors, as suggested in [23].

### 3.4. The Stalling Effect on GS Performance

**Figure 1:**
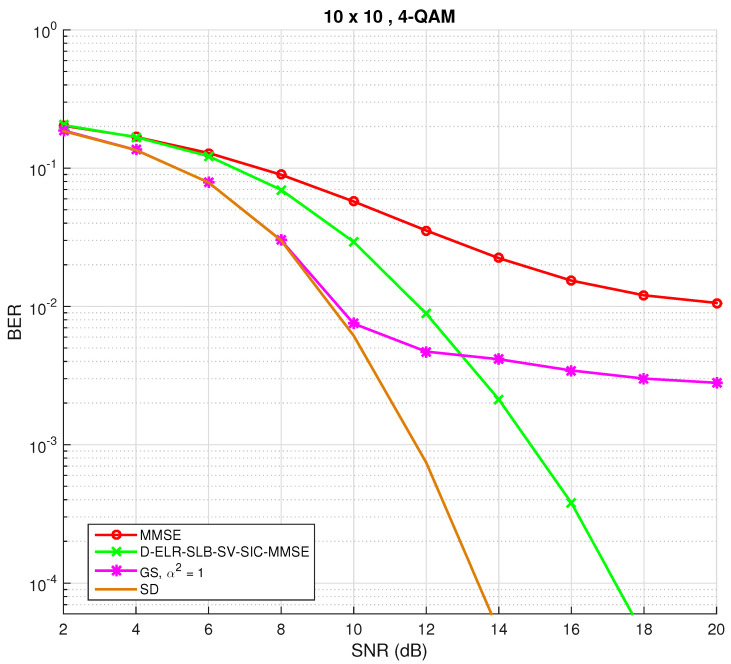
BER performance for conventional Gibbs sampling detection algorithm for M=N=10 and a 4-QAM constellation. A simulated performance of the D-ELR-SLB-SV-SIC-MMSE [14] is also plotted for benchmarking.

The performance of the GS method shall now be evaluated. To this purpose, set the maximum number of block iterations $t_{\max}=500$ and the “temperature” parameter equal to the noise variance, that is $\alpha^{2}=1$. For a symmetric system with n=20 and a 4-QAM constellation, the corresponding BER plot is shown in Figure 1.

For comparison purposes, performance of linear MMSE filtering and also the dual-element-based lattice-reduction shortest-longest-basis with sorted-variance successive-interference-cancellation with MMSE (D-ELR-SLB-SV-SIC-MMSE), proposed in [14], are also depicted. This latter detector remains as one of the best low-complexity detection techniques for large symmetric MIMO.

As can be inferred for low SNR, the performance is very close to optimal. However, in the medium to high SNR, the BER did not improve any further. This problem is referred in the literature as the “stalling problem” [33]. This effect can be explained as follows: consider a case where for a candidate vector $x^{\left(t\right)}$, the norm $||y-\bar{H}x^{\left(t\right)}\left|\right|$ is significantly smaller than $||y-\bar{H}x_{j|\tilde{\omega }}^{\left(t\right)}\left|\right|$, where $x_{j|\tilde{\omega }}^{\left(t\right)}$ is the same $x^{\left(t\right)}$ with *j*th position changed with $\tilde{\omega }$. In this situation and, especially when SNR is high, one may find that after sampling $P(x^{\left(t+1\right)}=x^{\left(t\right)}|y,H)\approx 1$ and $P(x^{\left(t+1\right)}=x_{j|\tilde{\omega }}^{\left(t\right)}|y,H)\approx 0$ [33]. Thus, the conventional Gibbs sampler may remain stuck in the same state for a very long time and that effect consequence is depicted in Figure 1, as the number of iterations was fixed to $t_{\max}=500$. All in all and even though the chain is guaranteed to converge asymptotically when t→∞ [31], stalling may occur. A possible solution to alleviate this problem is studied next.

### 3.5. Mixed Gibbs Sampling with Multiple Restarts

As stated previously, stalling occurs because the ML cost of the state vector may be trapped in a local minima for many iterations [23]. That is in part due to the norm inside the exponential in (5), which may cause disparate sampling probabilities. One possible approach is to try and optimize the temperature parameter α such that the probability of encountering the optimal solution is as high as possible. This evaluation was done in [22], and its performance will be shown in a subsequent section. Following what was done in [23,27], a mixed Gibbs sampling will be studied. This consists of a change in the update rule in (8): instead of drawing *all* samples from (6) with one probability, there is a chance $q_{\infty }=\left(1-q_{1}\right)$ that the values are instead taken from a uniform distribution as

(13) $$p\left(x_{j}^{\left(t+1\right)}=\omega |\theta \right)∼U\left[0,1\right],$$

such that $\sum p(x_{j}^{\left(t+1\right)}=\omega |\theta )=1$. This corresponds to setting α=∞ in (6). Hence, the method acquires the name of mixed Gibbs sampling (MGS), and as long as $q_{\infty }$ is chosen accordingly, the stalling problem is alleviated: if stuck in a local minima, there is a non-zero probability $q_{\infty }$ that the detector performs a random walk to move away from the local solution. Unless otherwise stated, the mixing ratio value is set to $q_{\infty }=\frac{1}{n}$ (as suggested in [23]), which is equivalent to performing one random walk per each block iteration on average.

To further improve performance, the multiple restarts (MR) technique, as proposed in [23,27], will also be contemplated. Due to the random nature of MGS, different instances of the algorithm may lead to different outputs, even when the input values are the same. Denote the maximum number of allowed restarts by *R*. When R>1, the output solution is the one yielding the lowest ML cost and since each of the restarts is independent of the others, a parallel computation of the various instances can be performed to decrease overall detection time.

With the introduction of MR, the algorithm has now three distinct loop levels: *R* restarts, *t* block iterations and *n* sampling processes per block iteration. Thus, the method can become computationally demanding. However, when a close enough solution to optimal has already been found, additional iterations may be redundant. Hence, in order to limit complexity, a suitable stopping criterion can be defined.

If the ML cost remains unchanged for two consecutive block iterations, stalling is said to have occurred [23]. Then, stalling mode is entered, where only a maximum number of iterations $\theta_{s}$ more is allowed. If during stalling mode, a vector x with lower ML cost is found, then stalling mode is left; otherwise, the algorithm terminates after $\theta_{s}$ iterations. The parameter $\theta_{s}$ is chosen as large if the stalled ML cost is high and chosen as small when the margin for improvement is narrow. If the algorithm does not stop as a result of the stalling mode, then the initially set maximum number of block iterations $t_{\max}$ are performed.

Under the considered model and when x is error free, the ML cost is ${\left||n|\right|}^{2}$, whose distribution is a chi-square with $2N_{R}$ degrees of freedom, mean $N_{R}\sigma_{n}^{2}$ and variance $N_{R}\sigma_{n}^{4}$ [34] (Chapter 11). Thus, a normalized ML cost of solution vector x can be characterized as [23]

(14) $$\phi \left(x\right)=\frac{||y-{qHx||}^{2}-N\sigma_{n}^{2}}{\sqrt{N}\sigma_{n}^{2}}.$$

The metric (14) can be seen as a a difference between ML cost and the mean of ${\left||n|\right|}^{2}$ normalized by the standard deviation of ${\left||n|\right|}^{2}$ (note that under the channel model in (1), $\sigma_{n}^{2}=1$). In addition to the ML cost, the stopping criteria $\theta_{s}$ should also take into account the size of the QAM constellation. Accordingly, [23] proposed the following:

(15) $$\theta_{s}\left(x\right)=\left\lceil \max\left(c_{\min},10\log_{2}M\exp\left(\phi \left(x\right)\right)\right)\right\rceil ,$$

where $c_{min}$ is the minimum number of iterations following a stalling event and M is the constellation size, as defined in Section 2 (and here made equal for all dimensions).

Likewise, a stopping criteria for the maximum number of repetitions *R* can be defined. In a similar fashion to the previous case (see [23] for further insight), the algorithm stops if the number of restarts performed so far is greater than the following integer threshold:

(16) $$P=\lfloor \max\left(0,0.5\log_{2}M\phi \left(x\right)\right)\rfloor +1.$$

The usage of both MGS and the stopping criteria in (15) and (16) will provide close to optimal solutions, while yielding limited complexity, as will be verified in the next subsection.

### 3.6. Performance of Mixed GS with Multiple Restarts

**Figure 2:**
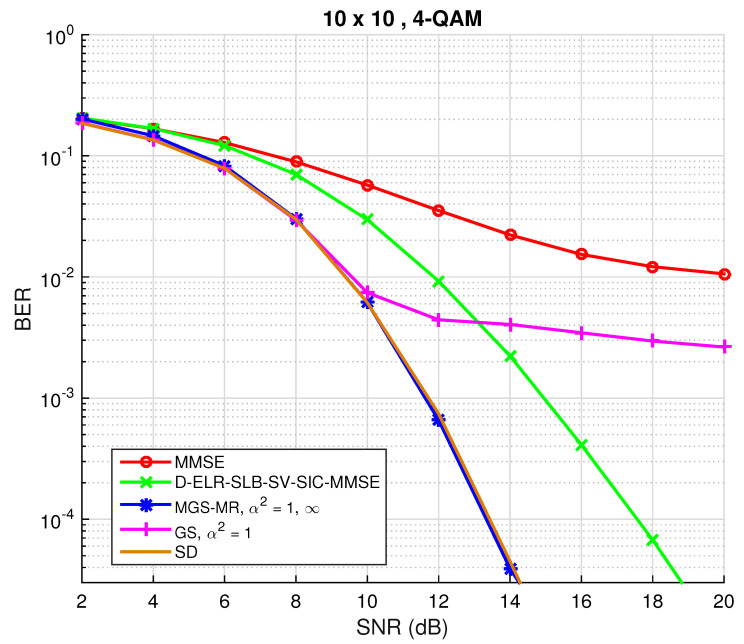
BER performance for both conventional Gibbs sampling and mixed Gibbs sampling with multiple restarts detection algorithms for M=N=10 and a 4-QAM constellation.

The performance of the described mixed Gibbs sampling with restarts will be evaluated here. Setting the number of dimensions n=20, the maximum number of restarts being R=15, the maximum number of iterations $t_{\max}=160$ and $c_{\min}=10$, the results are depicted in Figure 2. The initial vector for the first restart was the output of the MMSE filter and for the remaining restarts a random initial vector was used. As can be inferred, the stalling problem was efficiently fixed with the use of MGS-MR. Further, and as was expected, the attained diversity by MGS-MR is similar to the one obtained by the LRA method. Moreover, the BER gain attained by GS-MR over D-ELR-SLB-SV-SIC-MMSE is not negligible. In particular, the randomized algorithm requires around less 3 dB than the studied LR algorithm to achieve a BER of $10^{-3}$.

**Figure 3:**
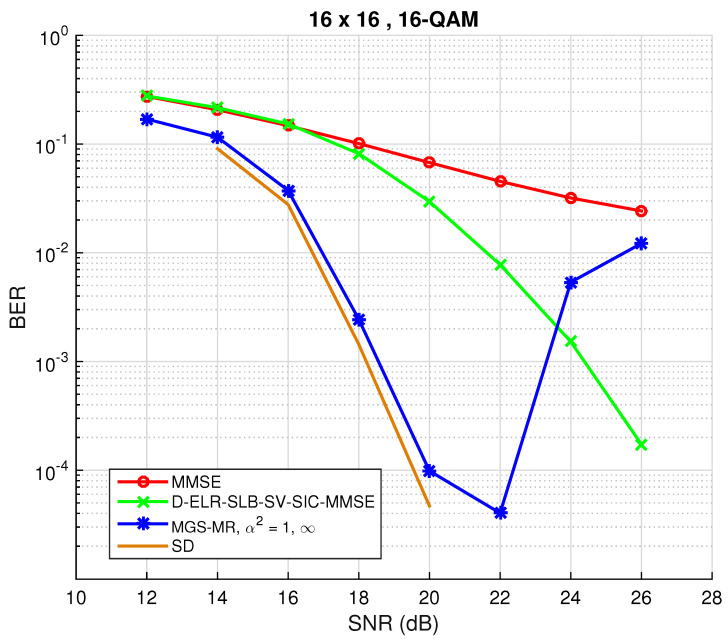
BER performance for both conventional Gibbs sampling and mixed Gibbs sampling with multiple restarts detection algorithms for M=N=16 and 16-QAM constellation.

Increasing dimensions to n=32, using a 16-QAM constellation and setting $t_{\max}=512$, the results are shown in Figure 3. As was expected, in the low SNR regime, the performance is near ML and better than the one achieved by the LRA method. However, for SNR ≥ 20 dB, the MCMC detector suffers a degradation in performance. This phenomenon is related with the rather low convergence rate (mixing time) of the underlying Markov chains, since $\alpha^{2}=1$. Particularly, this choice for α will cause the Markov chain to take a long time to reach its stationary distribution [22]. To solve this problem, three different approaches are possible:

1. Try and optimize the mixing ratio $q_{\infty }$ as a function of the SNR.
2. Find the dependence of α with the value of the SNR.
3. Increase the maximum number of iterations $t_{\max}$ and use different, better initial vectors $x^{\left(0\right)}$, at the cost of increased complexity.

Option 2 from the above will be evaluated next.

## 4. Optimization of the Temperature Parameter

Performance of GS based methods are highly dependent on the chosen TP. If $\alpha^{2}$ is set to equal the noise variance (that is $\alpha^{2}=1$), as in the conventional GS, a fast convergence to a local minimum is guaranteed but an error floor in the high SNR regime is to be expected. This is related with the existence of several local minima where the algorithm may be stuck for a very long time, a situation that becomes increasingly preponderant when either the $m_{k}$-arity or *n* are large. On the other hand, if $\alpha^{2}$ is chosen large, convergence rates of the underlying Markov chain increase (faster mixing time), but the likelihood of visiting the optimal solution in the steady state decreases, hence resulting in a trade-off situation in the choice of the temperature parameter.

In order to overcome this problem, the authors in [23] proposed a mixed GS (MGS) where at each iteration there is a small chance that a random walk is performed (which coincides with setting $\alpha^{2}=\infty$ in (6)). However, this method proves to mix slowly to the steady state, hence requiring a considerable amount of restarts and iterations in order to perform reliable detection. On the other hand, ref. [22] proposed a method comprising of a fixed, optimized TP $\alpha_{\mathrm{opt}.}^{2}$, proportional to SNR, as follows:

(17) $$\alpha_{\mathrm{opt}.}^{2}=\frac{\mathrm{SNR}}{\log(n)}+\sqrt{{\left(\frac{\mathrm{SNR}}{\log(n)}\right)}^{2}-2\frac{\mathrm{SNR}}{\log(n)}},$$

only valid for SNR>2logn, as one is interested in. This corresponds to the largest possible TP, such that, once in the stationary distribution, there is polynomially small and not exponentially small chance of finding the optimal vector. The method using the fixed TP as in (17) will henceforth be denoted as optimized GS (OGS). Notwithstanding the breakthrough developments, the results in [22] were only obtained for a {−1,1} constellation set, which is rather limited. It is interesting to note that the result in (17) suggests that $\alpha_{\mathrm{opt}.}$ scales with $O(\sqrt{\mathrm{SNR}})$, for fixed *n*, which means that faster mixing times are required in the high SNR regime.

**Figure 4:**
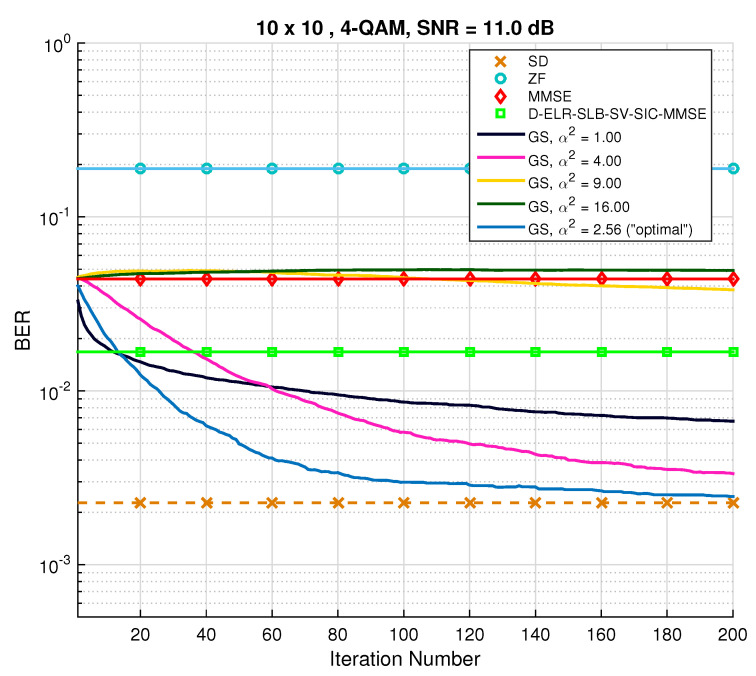
BER performance for conventional Gibbs sampling with different “temperature” parameters. M=M=10 and a 4-QAM constellation were used.

**Figure 5:**
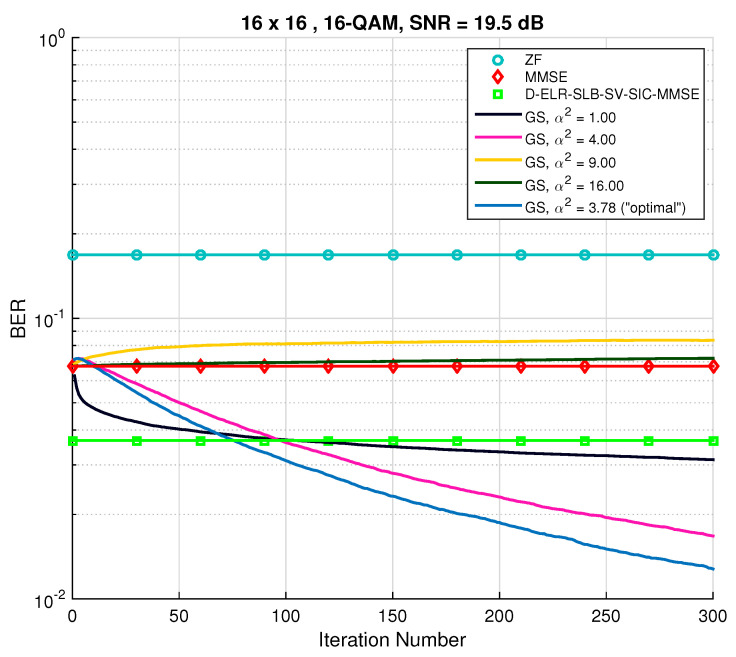
BER performance for conventional Gibbs sampling with different “temperature” parameters. M=N=16 and a 16-QAM constellation were used.

In order to demonstrate that MCMC methods employing Gibbs sampling converge to the optimal solution and that the parameter α plays a crucial role in that convergence, a study of BER evolution in terms of the number of iterations will next be entailed. For that purpose, the SNR was fixed, and a set of values for $\alpha^{2}$ was chosen, including $\alpha^{2}=1$ and $\alpha^{2}=\alpha_{\mathrm{opt}.}^{2}$. Using the MMSE filter output as the initial vector, Figure 4 and Figure 5 show the change in BER with an increasing number of iterations. For comparison purposes, conventional detectors are also depicted. The first thing to be noticed in Figure 4 is the fast convergence of the GS method to the ML performance when $\alpha^{2}=“\mathrm{optimal}”$ (blue line). Moreover, it is interesting to observe the rapid BER decay in the first few iterations for when $\alpha^{2}=1$ and then the stalling effect, as had been previously verified. Higher choices of $\alpha^{2}$ (namely, $\alpha^{2}=9$ and $\alpha^{2}=18$) proved not to improve the MMSE initial solution any further.

**Figure 6:**
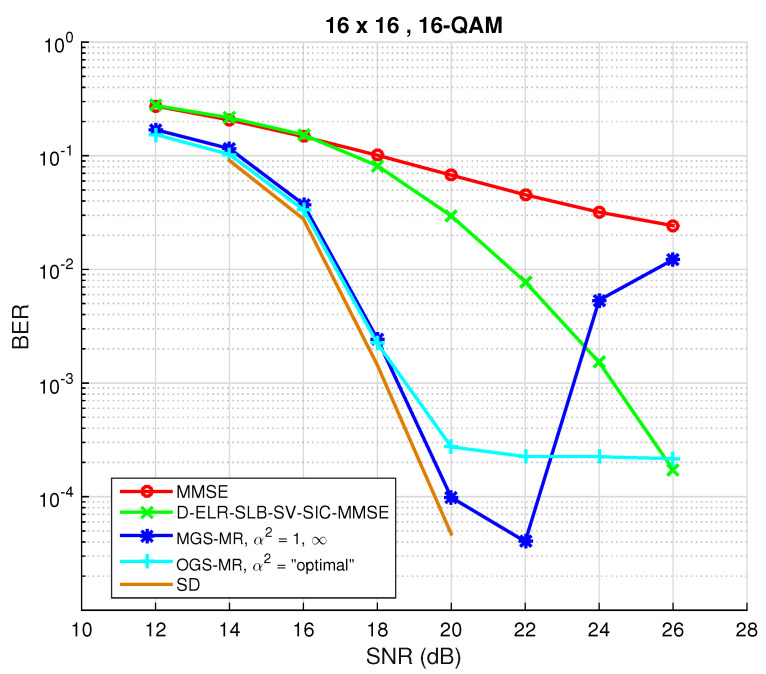
BER performance for optimized Gibbs sampling with multiple restarts (OGS-MR) detection algorithm for M=N=16 and a 16-QAM constellation.

Let one denote the detector using $\alpha^{2}=“\mathrm{optimal}”$, mixing ratio $q_{\infty }=0$ and multiple restarts, as optimized Gibbs sampling with multiple restarts (OGS-MR). The results corresponding to OGS-MR are depicted in Figure 6. For comparison purposes, the remaining used parameters are identical to the ones used to obtain Figure 3. As can be seen and as expected, the problem in the high SNR regime was partially mitigated, confirming that choosing an α different from 1 is indeed beneficent. Nonetheless, the stalling problem is present once again, and that is due to the existence of relative minimums, as explained in the comments regarding Figure 1.

Having in mind the results obtained so far, one can now present a novel combination of both MGS and OGS methods, which closes the gap in performance for large MIMO detection and for high SNR values.

## 5. Proposed Triple-Mixed Gibbs Sampling

Here, we propose an algorithm that at each iteration picks a different value for the TP from the set {∞,SNR/log(n),1} with probabilities $q_{\infty }$, $\left(1-q_{\infty }\right)q_{\mathrm{SNR}}$, and $\left(1-q_{\infty }\right)q_{1}$, respectively. In this manner, at each iteration there is a chance $q_{\infty }$ that a random walk is performed; otherwise, the algorithm chooses $\alpha^{2}=1$ or $\alpha^{2}=\mathrm{SNR}/\log\left(n\right)$ with probabilities $q_{1}$ and $q_{\mathrm{SNR}}$, and samples accordingly. Note that since we are interested only in the high SNR regime (SNR→∞) and to avoid dealing with imaginary TP, the term inside the square root operator has been despised. For each sampling process, choosing $\alpha^{2}=1$ ensures fast convergence to a local minimum, $\alpha^{2}\propto \mathrm{SNR}$ guarantees a quick mix to steady state and $\alpha^{2}=\infty$ is necessary to avoid “deep” local minima. Whilst yielding the same complexity, we find that with a suitable choice for $q_{\infty }$ and $q_{1}$ ($q_{\mathrm{SNR}}$ is redundant, as $q_{1}+q_{\mathrm{SNR}}=1$), it is possible to effectively mitigate the stalling effect and achieve near-ML performance. The pseudocode related with the proposed variant, denoted as triple MGS (T-MGS), is presented in Algorithm 2, where U[0,1] denotes the uniform distribution between 0 and 1, and pmf stands for probability mass function.

### Mixing Ratio Choice

**Figure 7:**
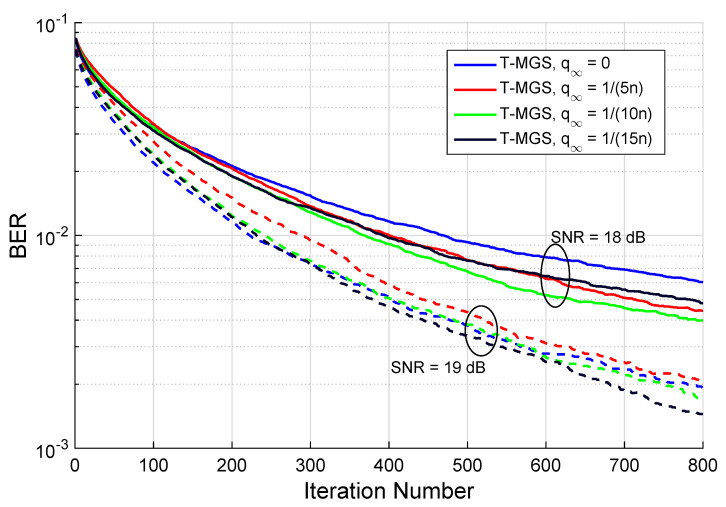
BER performance as a function of the number of iterations for different mixing ratios $q_{\infty }$ (with one restart only, and the MMSE solution was used as the initial vector). The simulations are for M=N=16 (i.e., n=32) with a 16-QAM constellation.

For demonstration purposes, consider n=32, $m_{k}=16$, for k=1,…,n, and $q_{1}=q_{\mathrm{SNR}}=1/2$. The performance in terms of BER as a function of the number of iterations for different mixing ratios $q_{\infty }$ is depicted in Figure 7, where two different SNR values were considered (18 and 19 dB). The first thing to be inferred, after the $t_{\max}=800$ iterations, is that in both cases it is a non-zero mixing ratio ($q_{\infty }\neq 0$) that minimizes the BER, hence proving the advantage of having a triple MGS algorithm. Additionally, the minimum value for different SNR values is attained for different values of $q_{\infty }$, implying that the optimal value of $q_{\infty }$ also has a dependence with the SNR.
**Algorithm 2.** Triple mixed Gibbs sampling algorithm with multiple restarts and stopping criteria.**Input:** $y,H,x^{\left(0\right)},t_{\max},q_{\infty },q_{1},R$Define:f(x)→ ML cost function (Equation (7))ϕ(x)→ Normalized ML cost function (Equation (14))$\theta_{s}\left(x\right)\to$ Stalling limit function (Equation (15))p(x,α|θ)→ Transition probability distribution (Equation (6))$c_{b}\to$ Cost of best candidate so far; set initial $c_{b}$ to *∞*Compute:**for**r=1 to *R* **do**    Initial candidate: $z=x^{\left(0\right)}$; Initial cost: β=f(z)    t=0    **while** $t<t_{\max}$ **do**        **for** i=1 to *n* **do**           generate $r_{1},r_{2}∼U\left[0,1\right]$           **if** $r_{1}<q_{\infty }$ **then**               generate pmf $p\left(x_{i}^{\left(t+1\right)}=\omega \right)∼U\left[0,1\right],\forall \omega \in A$               sample $x_{i}^{\left(t+1\right)}$ from this pmf           **else**               **if** $r_{2}<q_{1}$ **then**                   $\alpha^{2}$ = 1               **else**                   $\alpha^{2}\propto \mathrm{SNR}$ (according to Equation (17) and Table 1)               **end if**               $x_{i}^{\left(t+1\right)}∼p\left(x_{i},\alpha |x_{1}^{\left(t+1\right)},\ldots ,x_{i-1}^{\left(t+1\right)},x_{i+1}^{\left(t\right)},\ldots ,x_{n}^{\left(t\right)}\right)$           **end if**        **end for**        $\gamma =f(x^{\left(t+1\right)})$        **if** γ≤β **then**           $z=x^{\left(t+1\right)}$; β=γ        **end if**        t=t+1; $\beta_{v}^{\left(t\right)}=\beta$        **if** $\beta_{v}^{\left(t\right)}==\beta_{v}^{\left(t-1\right)}$ **then**           compute $\theta_{s}\left(z\right)$           **if** $\theta_{s}<t$ **then**               **if** $\beta_{v}^{\left(t\right)}==\beta_{v}^{\left(t-\theta_{s}\right)}$ **then**                   **break while**               **end if**           **end if**        **end if**    **end while**    **if** $f\left(z\right)<c_{b}$ **then**        $z_{b}=z$; $c_{b}=f\left(z_{b}\right)$    **end if**    compute $P=\lfloor \max\left(0,0.5\log_{2}M\phi \left(z_{b}\right)\right)\rfloor +1$    **if** P<r **then**        **break for**    **end if****end for****Output:** $z_{b}$

Finding the optimal choice for the mixing ratios *q* is a non-trivial task; nevertheless, one is to find parameters that on average perform well for any SNR. The authors in [23] proposed the MGS method with $q_{\infty }=1/n,q_{1}=1$, whereas [22] suggested the OGS with $q_{\infty }=0,q_{\mathrm{SNR}}=1$. With the T-MGS algorithm, one expects to experience fewer stalling occurrences in local minima than in MGS, as long as $q_{\mathrm{SNR}}>0$ (faster mixing time). This fact was verified through simulation and, as depicted in Figure 7, a choice of $q_{\infty }=1/\left(10n\right),q_{1}=q_{\mathrm{SNR}}=1/2$ proved to attain very satisfactory results, the reason why these values will be adopted henceforth in the paper for the T-MGS. Note that with this choice of parameters, one random walk is performed each 10 iterations on average, and whenever it is not carried out, there is a similar chance that $\alpha^{2}=1$ or $\alpha^{2}\propto \mathrm{SNR}$ are chosen.

A step-by-step description of the proposed algorithm including multiple restarts can be found in Algorithm 2. It is worth mentioning that the previously mentioned MGS-MR corresponds to doing $q_{\infty }=1/n$ and $q_{1}=1$, whilst OGS-MR coincides with setting $q_{\infty }=q_{1}=0$ in Algorithm 2. The “temperature” parameters and corresponding mixing ratios are summarized in Table 1.

**Figure 8:**
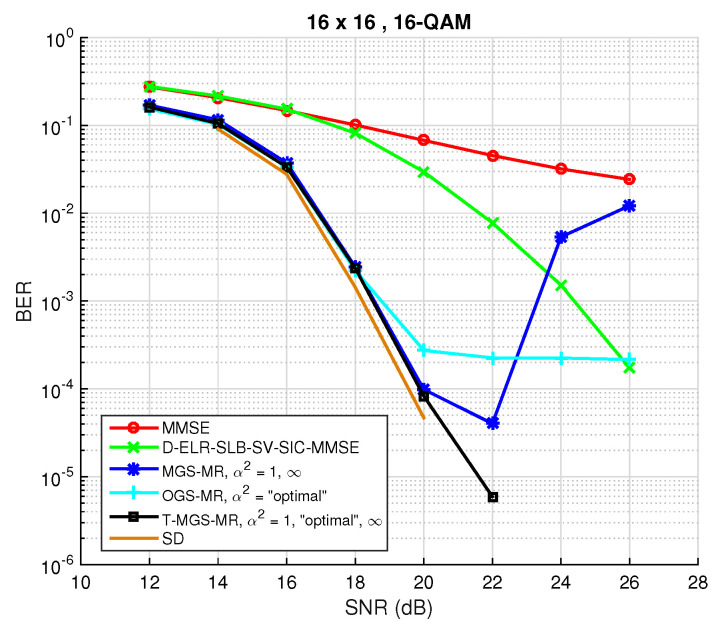
BER performance as a function of the SNR for the optimized, mixed and triple mixed Gibbs sampling algorithms with multiple restarts for $N_{R}=N_{T}=16$ and a 16-QAM constellation, using R=15 restarts.

Reconsidering the case where R=15 and the stopping criteria as was done for Figure 3 and Figure 6, the BER performance including the T-MGS detector is shown in Figure 8. The previously identified problems have been mitigated by T-MGS, and the curve in Figure 8 shows that optimal diversity is indeed obtained for the entirety of the studied SNR range. Moreover, for a reference BER value of $10^{-3}$, a gain of 5 dB is attained by the proposed algorithm over the state-of-the-art LRA method (the green curve). Taking into account the previously mentioned complexity reduction techniques and the attained performance, T-MGS is certainly a suitable candidate for solving the detection problem in large MIMO systems. In addition, it is worth emphasizing that the algorithm is parallel architecture-friendly, which is nowadays becoming an increasingly important feature.

## 6. Adaptively Modulated MIMO Detection

In a MTC-dominated context, when a number of different (single-antenna) devices transmits data to a BS, they should not be all transmitting using the same modulation order. This can happen due to ad hoc deployment of terminals (such as sensors) or in a planned manner. In an AM-MIMO system, the various transmitters adjust their parameters (e.g., their transmit power, or modulation order [19]) according to the channel state information (CSI) in order to attain a target objective (e.g., data throughput, spectral efficiency). Since each antenna is affected by different channel conditions, this adjustment feature often leads to non-negligible gains when compared to the most often studied fixed MIMO.

In this paper, we focus on solving the problem of detection when the different users have dissimilar constellation sizes using the proposed GS method. In fact, since the sampling procedure in (6) is done sequentially and because each instance corresponds to a given user, the adaptation of Algorithm 2 is quite straightforward, and can be summarized as follows:

1. Resorting to the CSI, determine the constellation size of each user according to a given criteria (for instance, using the proposed scheme in [19]). Each user has then an assigned alphabet from which is allowed to transmit, denoted by $A_{k}$.
2. Apply Algorithm 2 to the received vector (1), taking into account that the construction of the sampling probabilities in (6) is user dependent, i.e., each candidate entry to be sampled can only be chosen from $A_{k}$.

To best of the authors’ knowledge, this is the first time that a detector based on a randomized algorithm is utilized to perform detection in AM-MIMO (alternatives in the literature include either linear detection [17] or SD-based methods [20], whose performance-complexity trade-offs render them useless in large dimensions). Further, given the random nature of the proposed detector, it can be easily extended to generate soft-output values [23], which are of paramount importance when coded transmissions are used.

## 7. Assessment of the Proposed GS for Large MIMO and AM-MIMO

**Figure 9:**
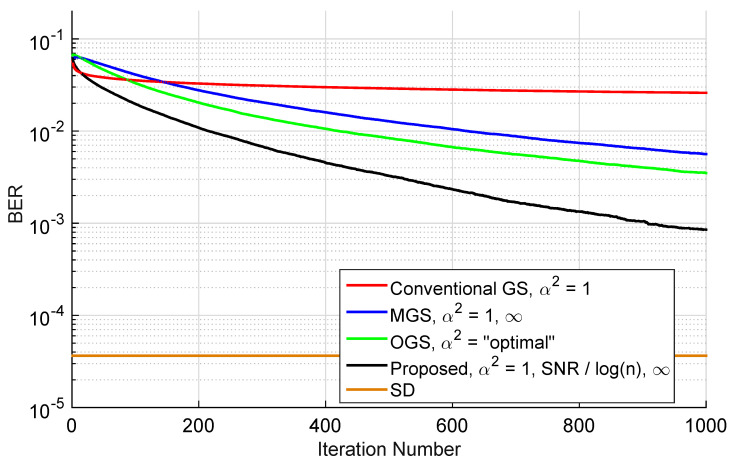
BER performance as a function of the number of iterations *t* for SNR = 20 dB for different TP options (not including MR). The simulations are for n=32, with a 16-QAM constellation.

In order to verify the superiority of T-MGS over MGS and OGS, a comparison of the average BER performance against the number of iterations is first provided in Figure 9 for symmetric MIMO. The number of antennas was set to N=16, a 16-QAM constellation was used, the number of iterations $t_{\max}$ was set to 1000, while the SNR was fixed to 20 dB. Additionally, the conventional GS method ($\alpha^{2}=1$) is shown, and the “optimal” TP parameter in the OGS method is computed according to [22] (Equation (23)). In all instances, the MMSE filter output was chosen as the initial candidate vector $x^{\left(0\right)}$. For comparison purposes, SD performance (which attains ML performance) is also depicted. As can be verified, different mixing ratios exhibit dissimilar behaviors. Firstly, it is confirmed that conventional GS has a rapid decay in BER in the first few iterations, but then becomes stalled in a local minima. Secondly, it is observed that the proposed method T-MGS outperforms both MGS and OGS by a non-negligible margin, thus showing the importance of choosing adequate mixing ratios. Nonetheless, the performance observed in Figure 9 tends to a BER that is still far from the optimal, which is achieved via SD, therefore proving the usefulness of executing MR, as suggested in [23,27], and whose effect will be analyzed next.

**Figure 10:**
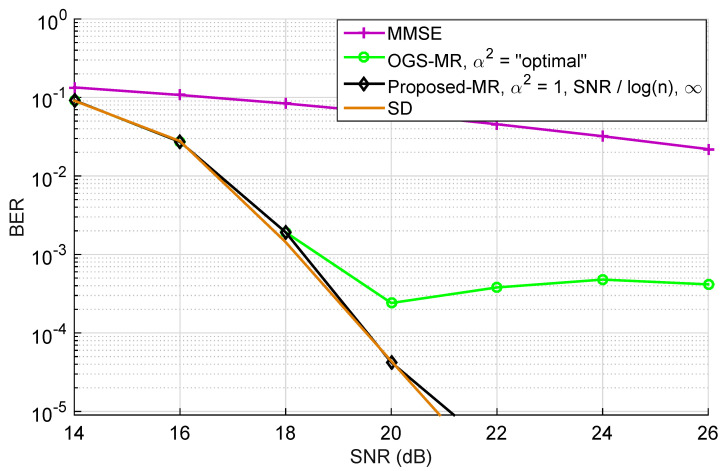
Comparison summary of the BER performance as a function of the SNR of the proposed variant for large MIMO systems. The simulations are for n=32, with a 16-QAM constellation (the system analysed in Figure 9, now with R=10).

For the assessment of BER as a function of the SNR, and taking into account the previous result, R=10 restarts are performed, fulfilling $t_{\max}=600$ iterations each, and the output coincides with the candidate vector yielding the lowest ML cost. For the first restart, the MMSE solution is used as the initial vector, and for the remaining ones, a randomly chosen candidate is picked from the lattice instead. The performance for these settings is depicted in Figure 10, which serves as a summary of the performance that the proposed T-MGS technique is able to achieve with a quite low complexity: in Figure 8, one only uses R=10 restarts, rather than the R=10 restarts in Figure 8, and the a BER and SNR are in the regions of practical interest. As one could have already seen in Figure 6 and Figure 8, a first thing to be noted is the existence of a stalling effect in OGS, which proves that [22] (Equation (23)) does not necessary scale for constellations larger than 4-QAM. Further, it can be verified that, with the inclusion of the MR approach, T-MGS performs very closely to the SD, whilst achieving near-optimal diversity for the whole studied SNR range. Again, it is worth emphasizing that the MR instances are parallel-architecture friendly, therefore such hardware design model, increasingly available at the BS, can be exploited to increase speed.

**Figure 11:**
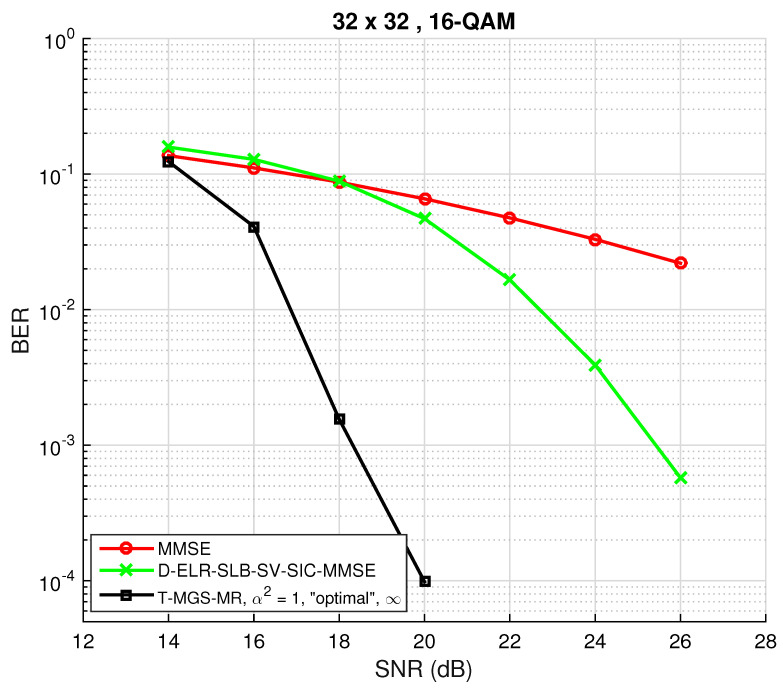
BER performance as a function of the SNR for the proposed triple mixed Gibbs sampling (T-MGS) algorithm with multiple restarts for $N_{R}=N_{T}=32$ and a 16-QAM constellation. The simulated performance of the LRA benchmark is also plotted.

In order to further confirm the suitability of the proposed algorithm for a quite large symmetric MIMO system (M=N=32), a simulation using $q_{\infty }=1/\left(10M\right)$, R=15 and $t_{max}=1000$ was performed. The result is depicted in Figure 11, and the previously obtained conclusions can also be applied here. Note that, further augmenting the number of antennas or the constellation size would lead to a drastic increase in complexity, even using the stopping criteria or the complexity reduction techniques suggested in Section 3.3. Nonetheless, the algorithm can be efficiently used to obtain an estimate of SD performance for any number of dimensions, provided the initial vector is close to the initial solution (for instance, feeding the solution of the D-ELR algorithm as the initial vector in T-MGS would lead to a lower number of average number of required iterations).

**Figure 12:**
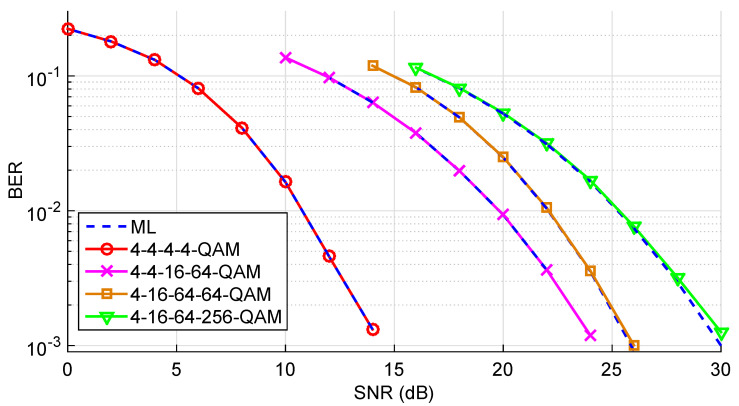
BER performance as a function of the SNR of T-MGS detection for AM-MIMO systems in a case with M=4 terminals, each of which using the modulations listed in the labels.

Finally, a comparative study is conducted between ML detection (using SD) and the T-MGS method for AM-MIMO systems employing M=4 terminals and N=4 antennas at the BS. The number of iterations $t_{\max}$ was set to 600, whereas the number of restarts was only R=5. The BER performance for four distinct sets of constellations is shown in Figure 12. It should be noted that, given the low-complexity and power limitations of MTC devices, these terminals are expected to transmit using low-order modulation in the uplink. Millimeter-waves (mmWaves) systems are on the rise, but those also tend to use low-order modulation due to both the high attenuation channels (that would imply larger transmit powers to keep the SNR) and the challenges posed to the analogue-to-digital converters (ADCs) when using wideband signaling. For these reasons, constellations larger than 16-QAM are uncommon in 5G mmWave systems [35]. Even so, the AM-MIMO system considered users with 4-QAM, 64-QAM, and 256-QAM. Once again, it can be verified the quasi-optimal performance of our algorithm, proving itself as a viable alternative over SD-based AM-MIMO detectors.

## 8. Conclusions

This paper proposed a new triple mixed Gibbs sampling algorithmic approach to perform detection in large MIMO and adaptively modulated MIMO systems. The latter systems appear as the most natural type of cell-based MIMO uplink when multiple MTC terminals exist and a massive MIMO BS is absent. The paper identifies the existing trade-offs in the choice of the temperature parameter, and capitalizing on those, a suitable selection for both mixing times and mixing ratios was presented. Numerical results show that the suggested variant outperforms the existing ones in the literature for symmetric large MIMO, whilst requiring a lower number of iterations. Further, exploiting the delay-less multiple restarts feature, the proposed variant is shown to obtain near-optimal performance. These results were obtained for large MIMO with full-loaded cells, rather then with loads $0.75<\frac{N}{M}<1$ as in the recent work [27]; even so, the attained performance by the proposed T-MGS is comparable to that one, when fairly comparing the performances at the same number of iterations.

Secondly, the paper shows how the proposed MIMO detection technique can be naturally extended to AM-MIMO, allowing us to detect in each MIMO stream a different alphabet, which is a feature that is not as straightforward to implement in other types of receivers, that often require a complicated control of the lattice boundaries. Because GS receivers do not operate in the lattice domain, but rather directly in the constellation domain, these uneven alphabets are simpler to track in the different streams.

A natural extension of this research should be the adaptation of the T-MGS detector to soft-metrics and to coded MIMO.

## Figures and Tables

**Table 1:** “Temperature” parameters and corresponding mixing ratios used to compare the various GS methods, including the proposed one, for N=16 and when a 16-QAM constellation size is used.

	Mixing Ratio	$q_{\infty }$ ($\alpha^{2}=\infty$)	$q_{1}$ ($\alpha^{2}=1$)	$q_{\mathrm{SNR}}$($\alpha^{2}=\alpha_{\mathrm{opt}.}^{2}$)
Variant				
MGS		$1/2N_{T}$	1	0
OGS		0	0	1
T-MGS		$1/20N_{T}$	1/2	1/2

## Data Availability

Data is contained within the article.

## Abbreviations

The following abbreviations are used in this manuscript:

AM-MIMO	Adaptively modulation MIMO
BER	Bit error rate
CVP	Closest vector problem
GS	Gibbs sampling
LRA	Lattice-reduction-aided
MCMC	Markov chain Monte Carlo
MGS	Mixed GS
MIMO	Multiple-input multiple-output
pmf	Probability mass function
SD	Sphere decoder
SNR	Signal-to-noise ratio
TP	Temperature parameter
